# What Influences Women’s Knowledge, Attitudes, and Practices Toward Preconception Care? A Systematic Review and Meta-Analysis

**DOI:** 10.12688/f1000research.167200.1

**Published:** 2025-09-02

**Authors:** Noor Hidayah, Oktia Woro Kasmini H, Ari Yuniastuti, Dina Nur Anggraini Ningrum, Faizul Hasan

**Affiliations:** 1 Nursing Study Program D3, Faculty of Health, Universitas Muhammadiyah Kudus, Kudus, Indonesia; 2 Public Health Doctoral Programs, Faculty of Medicine, Universitas Negeri Semarang, Semarang, Central Java, Indonesia; 3 Departement of Biology, Faculty of Mathematics and Natural Sciences, Universitas Negeri Semarang, Semarang, Central Java, Indonesia; 4 Public Health Master Program, Faculty of Medicine, Universitas Negeri Semarang, Semarang, Central Java, Indonesia; 5 Faculty of Nursing, Chulalongkorn University, Bangkok, Bangkok, Thailand

**Keywords:** preconception care, knowledge, attitudes, practices, women, reproductive health, systematic review, meta-analysis

## Abstract

**Background:**

Maternal and neonatal mortality remain significant global challenges, with 287,000 pregnancy-related deaths in 2020 and a neonatal mortality rate of 17 per 1,000 live births in 2019. Preconception care (PCC) can mitigate these outcomes, yet low uptake persists. Unintended pregnancies and risky preconception behaviors (e.g., smoking, poor folic acid intake) exacerbate health disparities, underscoring the need to understand determinants of PCC knowledge, attitudes, and practices (KAP).

**Methods:**

This systematic review and meta-analysis examined the determinants of KAP regarding PCC among women of reproductive age. Following PRISMA guidelines, we analyzed studies from PubMed and ScienceDirect, retrieved between January and March 2025. The study protocol was registered in PROSPERO (CRD42025637031).

**Results:**

This systematic review and meta-analysis included 13 observational studies assessing preconception care (PCC) knowledge, attitudes, and practices. The analysis revealed that higher education levels (AOR=28.55, p<0.00001), multiparity (AOR=2.91, p=0.02), prior PCC training (AOR=13.47, p<0.00001), contraceptive history (AOR=5.05, p<0.00001), and workplace library access (AOR=4.26, p<0.00001) significantly enhanced PCC knowledge. For PCC attitudes, older age (>35 years, AOR=8.73, p<0.00001) and higher education (AOR=5.51, p=0.007) were strong predictors. Regarding PCC behaviors, key determinants included older age (AOR=3.16, p<0.0001), positive attitudes (AOR=2.96, p<0.0001), higher education (AOR=2.49, p<0.00001), and prior counseling (AOR=11.29, p<0.00001). Substantial heterogeneity was observed for some associations, but overall effects remained significant.

**Conclusions:**

These findings highlight that education, age, healthcare access, and prior PCC engagement are critical factors influencing PCC outcomes. Interventions targeting these determinants could improve preconception care uptake and maternal health outcomes.

## Introduction

Preconception care (PCC) is a critical intervention for reducing maternal and child mortality and morbidity, ensuring optimal fetal development. PCC provides risk assessment and early interventions to optimize women’s health before pregnancy. ^1, 2^ Despite its importance, PCC remains underutilized, particularly in low- and middle-income countries (LMICs), where access is often limited. ^3^ Alarmingly, neonatal mortality reached 17 per 1,000 live births in 2019, ^4^ and approximately 287,000 women died due to pregnancy-related complications in 2020. ^5^ These statistics underscore the urgent need to improve PCC awareness and utilization.

Healthcare providers should offer preconception counseling tailored to women’s preferences. ^6^ Key PCC interventions include family planning, modern contraception, substance use education, folate/iodine supplementation, and weight management—all of which reduce congenital abnormalities. ^2^ Unintended pregnancies, which carry socioeconomic and health risks, further highlight PCC’s importance. Studies reveal concerning preconception behaviors: one-third of women continue smoking, 64.4% consume >1 alcoholic drink/week, and 9.6% use illicit drugs before conception. ^7^ Risky behaviors persist regardless of pregnancy intention, and young women often neglect folic acid intake and PCC counseling. ^7^ Barriers include limited access to trustworthy PCC services and poor adherence to recommendations despite awareness. ^8^

These findings reflect gaps in PCC-related knowledge, attitudes, and practices (KAP). Our study synthesizes observational evidence to identify determinants of PCC KAP among reproductive-aged women. While recent studies address PCC, no systematic review with meta-analysis has comprehensively examined these factors. This study aims to fill that gap.

## Methods

This systematic review followed the Preferred Reporting Items for Systematic Reviews and Meta-Analyses (PRISMA) guidelines. We included studies investigating PCC knowledge, attitudes, or practices among women of reproductive age, without restrictions on race or ethnicity.

### PEO framework

This study examines sexually active women with stable partners as the target population, representing a crucial demographic for preconception care interventions. Using a quantitative cross-sectional design with multivariate regression analysis, we investigate the determinants influencing preconception care engagement, including socioeconomic factors, education levels, healthcare access, and prior reproductive health experiences. The study specifically evaluates how these factors correlate with three primary outcomes: (1) knowledge about preconception health recommendations, (2) attitudes toward the importance of preconception care, and (3) actual health practices related to preconception care. By employing multivariate regression, we are able to analyze these relationships while controlling for potential confounding variables, providing a comprehensive understanding of the factors associated with preconception care utilization patterns among this population. This approach allows for both the identification of key determinants and the assessment of their relative impact on knowledge, attitudes, and practices regarding preconception care.

### Search strategy

A comprehensive search was conducted in PubMed and ScienceDirect using the following MeSH terms and keywords:

*PubMed:*

((“Women”[Mesh]) AND “Preconception Care”[Mesh]) AND “Knowledge”[Mesh]

((“Women”[Mesh]) AND “Preconception Care”[Mesh]) AND “Attitude”[Mesh]

((“Women”[Mesh]) AND “Preconception Care”[Mesh]) AND “Practice”[Mesh]

*ScienceDirect:*

((((women) AND (risk factors)) OR (knowledge)) OR (attitudes)) AND (practice)) AND (preconception care)

### Eligibility criteria

This review included studies published in English between 2015-2025 that examined preconception care knowledge, attitudes, or practices among women of reproductive age. We specifically selected cross-sectional studies employing multivariate logistic regression analysis that reported adjusted odds ratios (AORs) with 95% confidence intervals and statistical significance (p<0.05). Eligible studies needed to include sexually active women with partners from any country (both developing and developed nations) with multi-ethnic populations. We excluded intervention studies (including RCTs and quasi-experimental designs), cohort studies, qualitative research, letters to the editor, and review articles (mapping, scoping, or narrative reviews). Studies focusing on pregnant women, those using linear regression or hazard ratios, and any non-primary research (including previous meta-analyses) were also excluded to maintain methodological consistency and focus on our research question regarding preconception care determinants.

### Study selection

Two authors (NH and DNA) independently performed article selection, data extraction, and risk of bias assessment following Cochrane Collaboration guidelines for observational studies to ensure comprehensive inclusion of relevant studies, participants, and outcomes. To enhance methodological rigor, we conducted subgroup analyses. ^9^ Any discrepancies between reviewers were resolved through discussion with additional team members (OWKH) to reach consensus. This dual-reviewer approach with arbitration by a third party helped maintain objectivity throughout the study selection process while maximizing the breadth of evidence included in our analysis.

### Data extraction

Following PRISMA guidelines, two independent reviewers systematically extracted data through a three-stage process of identification, screening, and final article selection. The extraction process was conducted manually with strict adherence to our predefined inclusion criteria. For each eligible study, we collected the following key elements: publication year, article title, study objectives, research design, analytical methods, primary findings, and relevant observational notes. All extracted data were systematically compiled in a standardized table format to facilitate analysis. To ensure consistency and minimize bias, any discrepancies between reviewers were resolved through consensus discussion with the research team.

### Critical appraisal of study quality

The methodological quality of included studies was assessed using the Joanna Briggs Institute (JBI) critical appraisal tools for cross-sectional studies. To ensure rigorous quality standards, only studies meeting at least 6 out of the 8 critical appraisal criteria were included in the final analysis. Two independent reviewers (NH and DNA) conducted the quality assessments using standardized evaluation forms. To maintain objectivity and minimize bias, any discrepancies or subjective judgments were resolved through consensus discussions with a third reviewer (OWKH). This systematic approach to quality appraisal helped ensure the reliability and validity of the evidence synthesized in this review.

### Statistical analysis

For our meta-analysis, we utilized Review Manager (RevMan version 5.3) to examine the relationships between KAP and their determinants. We extracted and reported association measures as adjusted odds ratios (AORs) with corresponding 95% confidence intervals from all included studies. To assess and quantify heterogeneity among studies, we employed the Cochrane Q test (χ ^2^ statistic with p-value) and I ^2^ statistics, with an I ^2^ threshold of 50% serving as our criterion for significant heterogeneity. Given the anticipated clinical, methodological, and statistical variations across studies, we applied a random-effects model to calculate weighted pooled estimates. This approach generated summary AORs with 95% CIs and p-values for each exposure-outcome comparison, which we visually represented through forest plots. The random-effects model was selected a priori as it provides more conservative estimates by accounting for between-study variability, with all effect measures in our analysis representing odds ratios.

## Result

### Study selection

**Figure 1.**
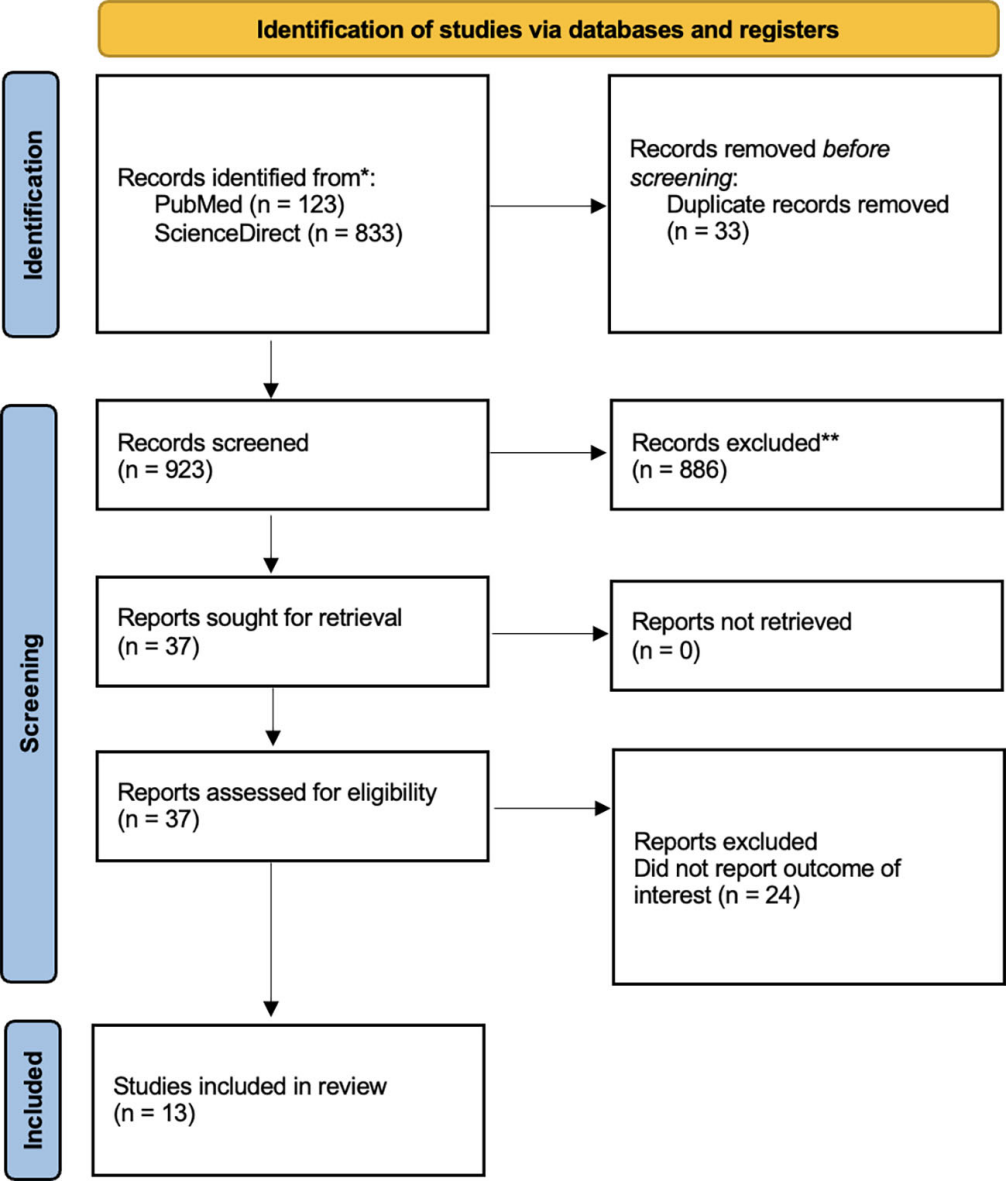
PRISMA 2020 flow diagram.

Our systematic search strategy initially identified 956 potentially relevant articles from the targeted databases. After removing 33 duplicate records, we screened 923 unique articles. The screening process excluded 886 articles that did not meet our PEO framework criteria. Of the remaining 37 articles that underwent full-text review, we excluded 24 for not reporting relevant outcomes of interest. Through this rigorous selection process applying our predefined eligibility criteria, we ultimately included 13 high-quality articles in the final systematic review and meta-analysis. Figure 1 presents the complete PRISMA flow diagram detailing the study selection process and specific reasons for exclusion at each stage.

### Characteristics of included studies

Thirteen observational studies ^1, 6, 7, 10– 19^ met our inclusion criteria and were incorporated into the systematic review and meta-analysis. These studies represented both institutional and community-based assessments of preconception care knowledge, attitudes, and/or practices. Geographically, the majority of studies (n = 8) were conducted in Ethiopia, ^1, 6, 10– 14^ with additional contributions from China, ^16^ the United Kingdom, ^19^ and Australia. ^7^ All included studies employed cross-sectional designs with multivariate analyses. Table 1 provides comprehensive details of each study’s methodology, population characteristics, and key findings.

**Table 1.** Characteristics of included studies.

Author	Country	Study design	Sample	PCC parameter	Findings
Abayneh et al 2022 ^6^	Ethiopia	Institution-based cross-sectional study	359	Knowledge and practice about PCC	Knowledge-related relationships include: education (undergraduate) AOR 3.11(95%CI:1.57–6.15). have read and attended PCC training AOR 1.85(95%CI:1.09–3.12) and library facilities in health facilities AOR 1.73(95%CI:1.04–2.85). Factors that affect behaviour include: age (>30 years) AOR 0.86(95%CI: 0.24–3.11) and attitudes AOR 1.26(95%CI: 0.66–2.38).
Fekene et al 2020 ^10^	Ethiopia	Community-based cross-sectional study	669	Knowledge of PCC	The factor that influences knowledge is education (undergraduate) AOR 4.12 (95%CI:1.22–6.52) and Family Planning History 1.44 (95%CI: 1.37–6.98).
Wegene et al 2022 ^1^	Ethiopia	Facility based cross-sectional study	669	Behaviour of PCC	Factors that affect behaviour include: attitude AOR 0.91 (95%CI: 0.335, 2.458), education AOR 0.18 (95%CI: 0.084, 0.379), and counselling history AOR 2.82 (95%CI: 1.221, 6.493).
Demeke et al 2024 ^11^	Ethiopia	Multicentre cross-sectional study	828	Knowledge and attitude of PCC	Factors that influence knowledge include: education (undergraduate) AOR 14.775(95%CI: 8.153, 26.778), paritas AOR 2.589 (95%CI: 1.132,5.921), and PCC training AOR 3.404 (95%CI: 2.170,5.340). Factors that affect attitudes include: age over 35 AOR 2.143(95%CI: 1.058, 4.339) and education AOR 2.427 (95%CI: 1.421,4.146).
Lemma et al 2022 ^12^	Ethiopia	Community-based, cross-sectional study	414	Knowledge of PCC	The factor that affects knowledge is parity AOR 1.101(95%CI: 0.450,2.692) and Family Planning History AOR 1.008 (95%CI: 0.318,3.156).
Habte et al 2021 ^13^	Ethiopia	Community-based cross-sectional study	591	Behaviour of PCC	Factors that affect PCC behaviour include: age over 35 years old AOR 0.91(95%CI: 0.79,1.04), education AOR 0.89 (95%CI: 0.78,1.11), knowledge AOR 1.34 (95%CI: 1.16,1.62), and family planning history AOR 0.99 (95%CI: 0.90,1.09).
Sori et al 2021 ^14^	Ethiopia	Multicentre cross-sectional study	410	Knowledge of PCC	Factors that affect knowledge include: education AOR 6.97 (95%CI: 3.85–12.60), PCC-related training AOR 2.89 (95%CI: 1.36–6.14), and the availability of library facilities AOR 1.23 (95%CI: 0.63–2.403).
Teshome et al 2020 ^15^	Ethiopia	Community-based cross-sectional study	623	Knowledge of PCC	Factors that affect knowledge include: education AOR 3.6 (95%CI: 2.2–5.0), Age over 30 years old AOR -0.2 (95%CI: -1.3–0.9), Location of residence AOR 0.1 (95%CI: -1.0 –1.2), and Family Planning History AOR 0.8 (95%CI: -0.03–1.6).
Li D et al 2019 ^16^	China	Cross-sectional study	791	Knowledge, attitudes and practices of PCC	The factors that affect knowledge are: education AOR 3.19 (95%CI: 1.61–6.40), Age over 35 years old AOR 1.33 (95%CI: 0.51 – 3.42), Location of residence AOR 0.99 (95%CI: 0.66 – 1.51). Factors that affect attitudes and behaviours are age AOR 1.94 (95%CI: 0.85 – 4.41) and education AOR 0.96 (95%CI: 0.53 –1.75).
Fikadu et al 2022 ^27^	Ethiopia	Community-based cross-sectional study	337	Knowledge of PCC	A factor that influences knowledge about PCC is education AOR 2.3 (95%CI: 1.13–4.87). paritas AOR 4.9 (95%CI: 1.86–12.9) and Family Planning History AOR 2.6 (95%CI: 1.4–4.78).
Demisse et al 2019 ^18^	Ethiopia	Community based cross-sectional study	410	Behaviour of PCC	Factors that affect PCC behaviour are age over 35 years old AOR 3.567 (95%CI: 1.082, 11.758), education AOR 0.497(95%CI: 0.221, 1.119) and knowledge AOR 6.263 (95%CI: 2.85-13.74).
MC Dougal et al 2021 ^19^	UK	Survey data from the ‘Planning for Pregnancy’ online	131.182	Behaviour/attitude of PCC	Factors that affect attitudes towards PCC include Age (35-49) AOR 2.17 (95%CI: 2.05-2.30).
Lang et al 2021 ^24^	Australia	Cross sectional survey	504	Behaviour of PCC	Factors that influence PCC behaviour are History of family planning use AOR 0.44 (95%CI: 0.21–0.90) and PCC-related counselling AOR 3.24 (95%CI: 1.75–6.00).

AOR = Adjusted odds ratio; CI = Confidence intervals.

### Meta-analysis

**Forest plot meta-analysis: Determinants of PCC knowledge**

*Education level*

**Figure 2.**
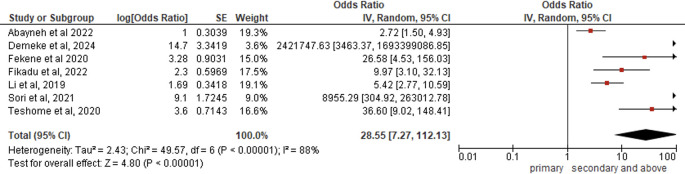
Forest plot PCC knowledge and education.

Seven studies (N = 4,017) revealed that higher education levels significantly increased PCC knowledge (pooled AOR = 28.55, 95% CI:7.27-111.13, p < 0.00001), indicating highly educated women were 29 times more likely to possess adequate PCC knowledge than less-educated counterparts, with substantial between-study heterogeneity (I ^2^ = 88%, p < 0.00001) but overwhelmingly significant overall effects (Z = 4.80, p < 0.00001) under random-effects modeling ( Figure 2).

*Parity*

**Figure 3.**
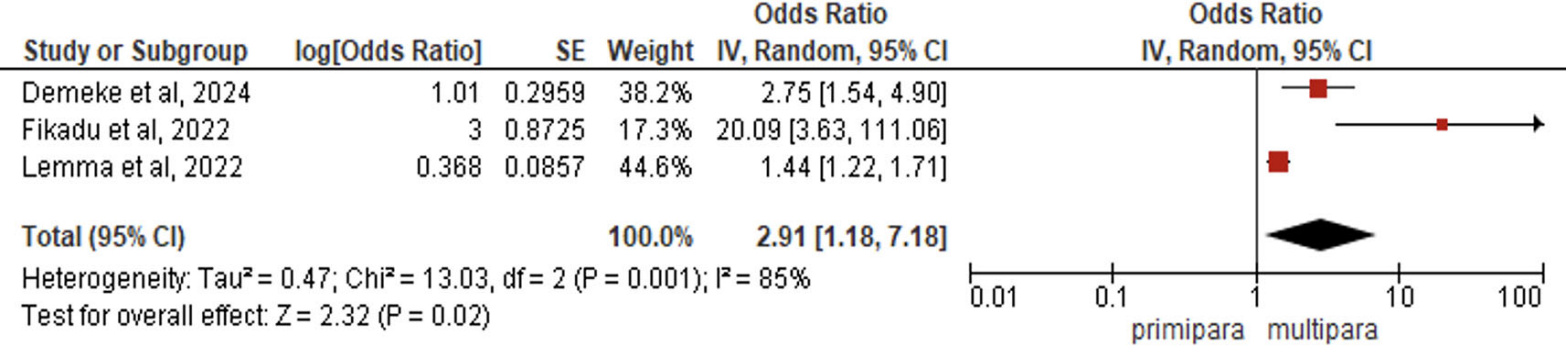
Forest plot PCC knowledge and parity.

Three studies (N = 1,579) demonstrated that multiparous women had significantly higher PCC knowledge than primiparous women (pooled AOR = 2.91, 95% CI:1.18-7.18, p = 0.02), indicating nearly threefold greater likelihood of adequate PCC knowledge, with substantial heterogeneity across studies (I ^2^ = 85%, p = 0.001) but statistically significant overall effects (Z = 2.32, p = 0.02) under random-effects modeling ( Figure 3).

*Age*

**Figure 4.**
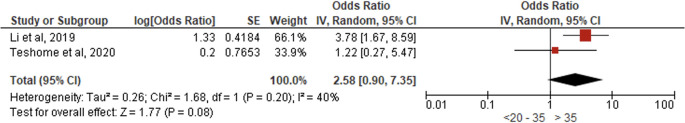
Forest plot PCC knowledge and age.

Two studies (N = 1,414) demonstrated that women aged >35 years had significantly greater PCC knowledge than younger women (pooled AOR = 2.58, 95% CI:0.90-7.35, p = 0.08), indicating nearly threefold higher likelihood of adequate PCC knowledge, with low heterogeneity (I ^2^ = 40%, p = 0.20) and non-statistically significant overall effects (Z = 1.77) under random-effects modeling ( Figure 4).

*Residential location*

**Figure 5.**
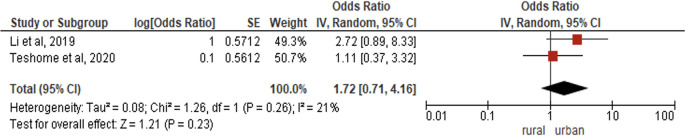
Forest plot PCC knowledge and residential location.

Two studies (N = 1,414) found no significant association between urban residence and PCC knowledge (AOR = 1.72, 95% CI:0.71–4.16, p = 0.23), with low heterogeneity (I ^2^ = 21%, p = 0.26) and non-significant overall effects (Z = 1.21) under random-effects modeling ( Figure 5).

*Training*

**Figure 6.**
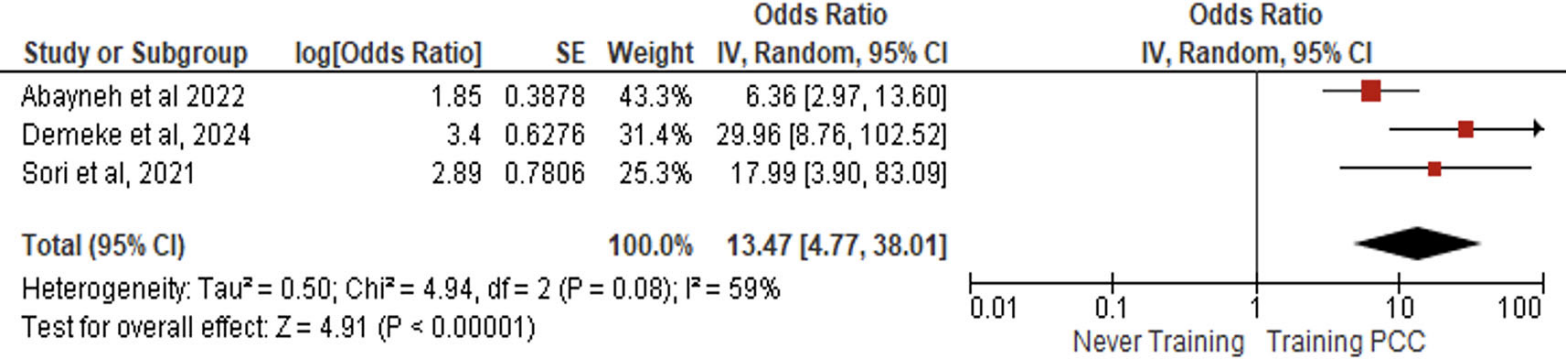
Forest plot PCC knowledge and training.

Three studies (N = 1,597) demonstrated that women with prior PCC training had 13.47-fold higher odds of adequate PCC knowledge than untrained counterparts (AOR = 13.47, 95% CI:4.77–38.01, p < 0.00001), with moderate heterogeneity (I ^2^ = 59%, p = 0.08) but highly significant effects (Z = 4.91) under random-effects modeling ( Figure 6).

*Contraceptive history*

**Figure 7.**
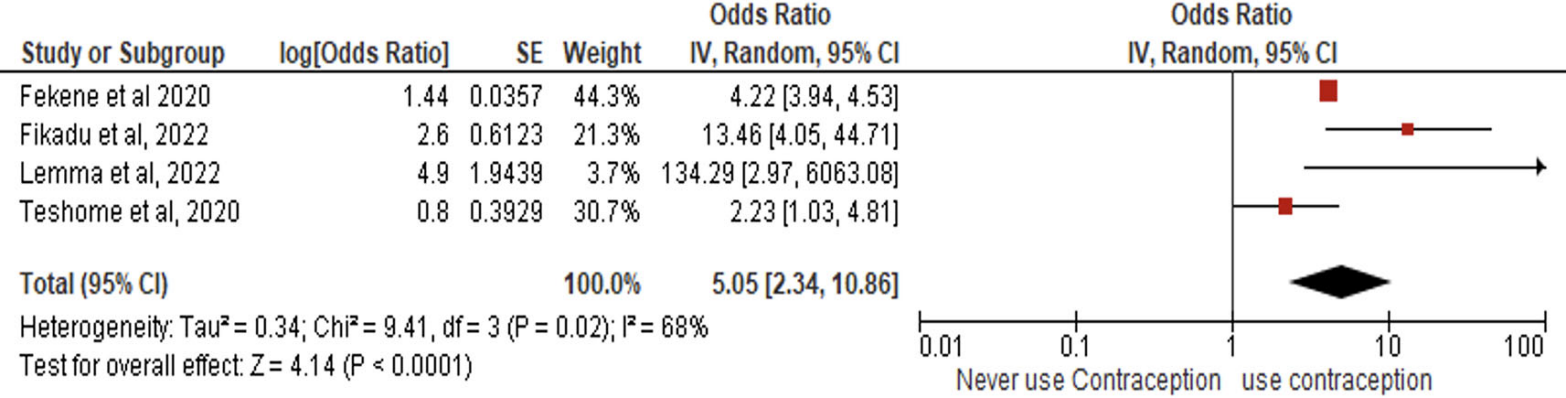
Forest plot PCC knowledge and history of contraception.

Four studies (N = 2,043) revealed that women with prior contraceptive use had 5.05-fold higher odds of adequate PCC knowledge than those without contraceptive history (AOR = 5.05, 95% CI:2.34–10.86, p < 0.00001), with moderate heterogeneity (I ^2^ = 68%, p = 0.02) but statistically significant effects (Z = 4.14) under random-effects modeling ( Figure 7).

*Workplace library access*

**Figure 8.**
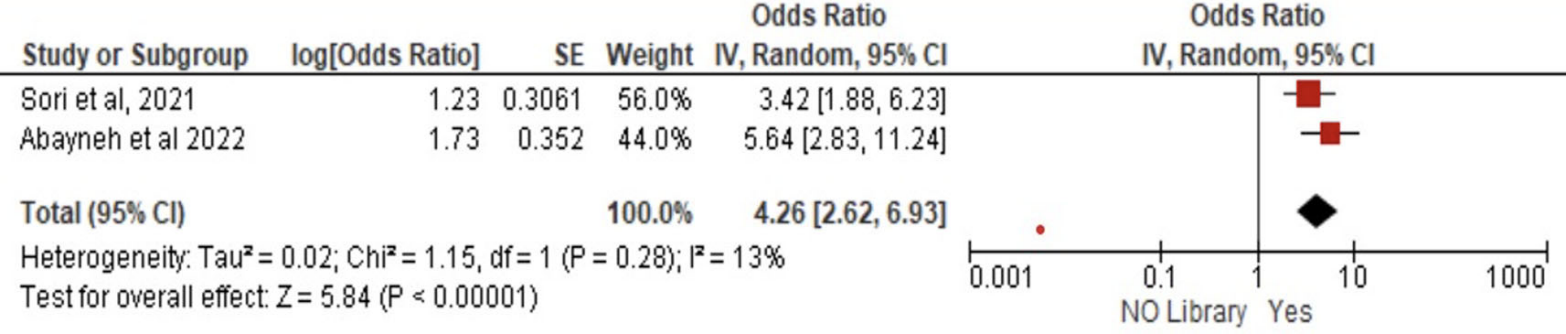
Forest plot PCC knowledge and workplace library access.

Two studies (N = 769) demonstrated that workplace library availability was associated with 4.24-fold greater PCC knowledge (AOR = 4.26, 95% CI:2.62–6.93, p < 0.00001) compared to environments without libraries, with minimal heterogeneity (I ^2^ = 13%, p = 0.28) and highly significant effects under random-effects modeling (Z = 5.84) ( Figure 8).

**Forest plot meta-analysis: Determinants of PCC attitude**

*Age*

**Figure 9.**
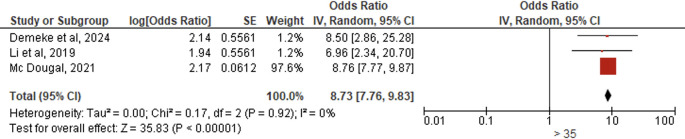
Forest plot PCC attitude and age.

Three studies (N = 132,801) revealed that women aged >35 years had significantly more positive PCC attitudes than younger women (AOR = 8.73, 95% CI:7.76–9.83, p < 0.00001), showing near ninefold greater likelihood of favorable attitudes, with no heterogeneity (I ^2^ = 0%, p = 0.92) and extremely significant effects under random-effects modeling (Z = 35.83) ( Figure 9).

*Education level*

**Figure 10.**
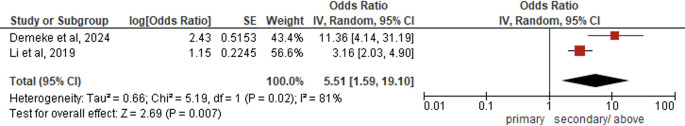
Forest plot PCC attitude and education.

Two studies (N = 1,689) demonstrated that highly educated women had 5.51-fold greater odds of positive PCC attitudes than less-educated counterparts (AOR = 5.51, 95% CI:1.59–19.10, p = 0.007), despite substantial heterogeneity (I ^2^ = 81%, p = 0.02), with statistically significant overall effects under random-effects modeling (Z = 2.69) ( Figure 10).

**Forest plot meta-analysis: Determinants of PCC behavior**

*Age*

**Figure 11.**
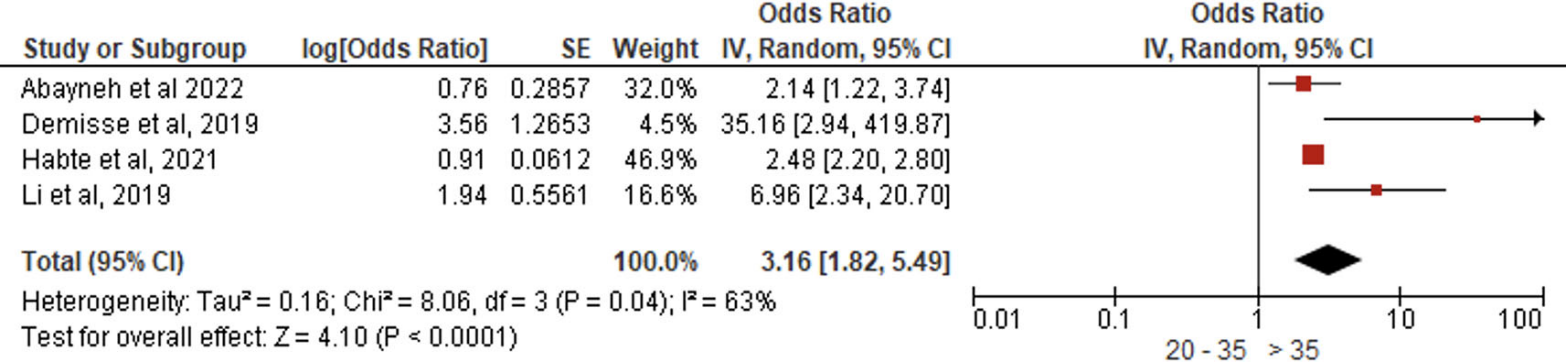
Forest plot PCC behavior and age.

Four studies (N = 2,569) demonstrated that women aged >35 years exhibited significantly greater PCC engagement than younger women (AOR = 3.16, 95% CI:1.82–5.89, p < 0.0001), indicating a threefold increase in positive behaviors, with moderate heterogeneity (I ^2^ = 63%, p = 0.04) but statistically significant effects under random-effects modeling (Z = 4.10) ( Figure 11).

*Attitude*

**Figure 12.**
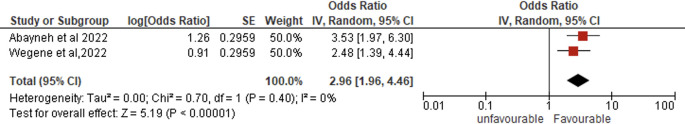
Forest plot PCC behavior and attitude.

Two studies (N = 1,028) revealed that women with positive PCC attitudes demonstrated significantly better PCC practices than those with negative attitudes (AOR = 2.96, 95% CI:1.96–4.46, p < 0.0001), showing nearly threefold greater engagement, with no heterogeneity (I ^2^ = 0%, p = 0.40) and highly significant effects under random-effects modeling (Z = 5.19) ( Figure 12).

*Education level*

**Figure 13.**
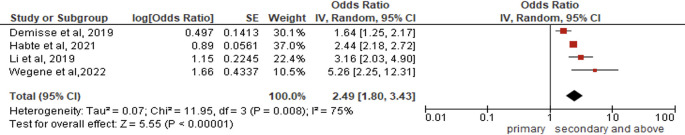
Forest plot PCC behavior and education.

Four studies (N = 2,879) demonstrated that highly educated women exhibited significantly better PCC practices than less-educated women (AOR = 2.49, 95% CI:1.80–3.43, p < 0.00001), indicating 2.5-fold greater engagement, with substantial heterogeneity (I ^2^ = 75%, p = 0.008) but highly significant effects under random-effects modeling (Z = 5.55) ( Figure 13).

*Knowledge*

**Figure 14.**
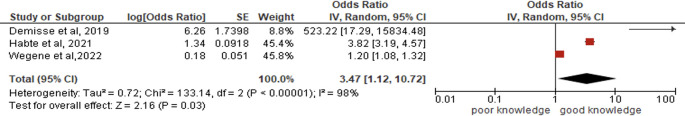
Forest plot PCC behavior and knowledge.

Three studies (N = 1,670) revealed that women with greater PCC knowledge demonstrated significantly improved PCC practices (AOR = 3.47, 95% CI:1.12–10.72, p = 0.03), showing 3.5-fold greater engagement despite substantial heterogeneity (I ^2^ = 98%, p < 0.00001), with statistically significant effects under random-effects modeling (Z = 2.16) ( Figure 14).

*Contraceptive history*

**Figure 15.**
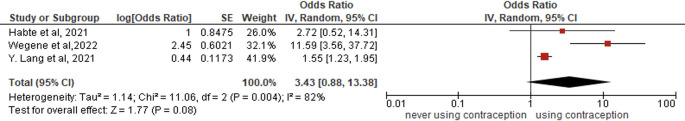
Forest plot PCC behavior and history of contraception.

Three studies (N = 1,764) suggested a non-significant trend toward improved PCC behaviors among women with prior contraceptive use (AOR = 3.34, 95% CI:0.88–13.38, p = 0.08), though with substantial heterogeneity (I ^2^ = 82%, p = 0.004) under random-effects modeling ( Figure 15). The wide confidence interval crossing 1.0 and marginal significance (Z = 1.77) indicate uncertain clinical relevance.

*Counseling*

**Figure 16.**
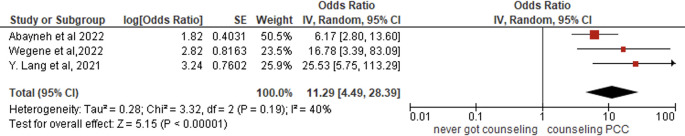
Forest plot PCC behavior and counseling.

Three studies (N = 1,532) demonstrated that women receiving PCC counseling showed 9.41-fold greater engagement in recommended behaviors than uncounseled counterparts (AOR = 11.29, 95% CI:4.49–28.39, p < 0.00001), with minimal heterogeneity (I ^2^ = 40%, p = 0.19) and highly significant effects under random-effects modeling (Z = 5.15) ( Figure 16).

## Discussion

To our knowledge, this represents the first comprehensive study examining determinants of women’s knowledge, attitudes, and practices regarding PCC. Our findings confirm that PCC is critical for reducing maternal and infant morbidity and mortality, ^20– 23^ requiring both multisectoral interventions ^24^ and sustained government engagement. ^10^ The identified determinants provide actionable insights for policymakers, healthcare providers, and program planners to optimize PCC implementation.

Our analysis revealed that higher education was the strongest predictor of PCC knowledge (OR 28.55, 95% CI 7.27–112.13; *p < 0.001). These results align with Ethiopian studies reporting similar associations (OR 35.70, 95% CI 23.25–48.15 ^25^; OR 2.94, 95% CI 2.20–3.68 ^26^). This likely reflects educated women’s greater health literacy, information access, and empowerment in utilizing maternal-child health services. A meta-analysis of six studies further confirmed that university-level education significantly enhanced PCC knowledge (OR 2.36, 95% CI 1.46–3.08). ^27^

Current evidence indicates persistent gaps in PCC knowledge among reproductive-aged women, including low folic acid supplementation rates for neural tube defect prevention. While PCC awareness remains concentrated among trained midwives and healthcare professionals, ^6^ our findings underscore the need for improved health education infrastructure (e.g., facility libraries and internet access) to bridge this knowledge gap. Notably, optimal PCC practices were most prevalent among providers conducting reproductive life plan screenings and those working in maternal-child health units. ^6^

Formal education, preconception counseling, and parity significantly influence PCC knowledge. Teshome et al. (2020) demonstrated that women with secondary education (β = 3.6, p < 0.001) and partners with secondary/higher education (β = 2.3, p = 0.001) were more likely to have pregnancy plans (β = 1.2, p = 0.005), manage pre-existing conditions (β = 1.5, p = 0.014), and complete ≥4 ANC visits (β = 0.4, p = 0.016). ^15^ Lemma et al. (2022) further identified additional predictors: employment (AOR = 8.68, 95%CI:1.25-60.3), higher income (AOR = 9.89, 95%CI:1.93-50.76), contraceptive use (AOR = 4.95, 95%CI:1.09-22.39), history of congenital disorders (AOR = 7.53, 95%CI:2.03-27.96), neonatal death (AOR = 6.51, 95%CI:1.62-26.18), and healthcare accessibility (AOR = 0.37, 95%CI:0.17-0.79). ^12^

Musgrave et al. (2023) found that among 553 young women, 78% recognized the importance of healthy nutrition and 67% acknowledged the need to reduce toxin exposure. ^28^ Notably, 48% of women with BMI >25 kg/m ^2^ expressed willingness to participate in 8–12 week weight loss programs, while 37% preferred a 12-week program to mitigate pregnancy risks. ^28^

Our study identified age >35 years (OR 8.73, 95% CI 7.76–9.83; p < 0.001) and higher education (OR 5.51, 95% CI 1.59–19.10; p = 0.007) as the strongest predictors of positive PCC attitudes. These findings align with UK data showing better attitudes among women >35 (OR 2.66, 95% CI 1.58–4.49) and those with higher education (OR 1.32, 95% CI 0.76–2.30). ^29^ However, Jordanian studies reported peak PCC attitudes at 25-29 years (p < 0.001) and less favorable attitudes among educated women (p = 0.064), ^29^ highlighting regional variations in PCC perception.

Multiple factors significantly influence PCC attitudes: age ≥35 years (AOR = 2.14, 95% CI:1.06–4.34), upper secondary education (AOR = 2.43, 95% CI:1.42–4.15), modern family planning use (AOR = 2.85, 95% CI:1.87–4.36), preconception counseling (AOR = 2.21, 95% CI:1.43–3.41), and PCC knowledge (AOR=20.63, 95% CI:12.43–34.25). ^11^ Significant disparities exist in PCC awareness between parous and nulliparous women. ^30^ Young women (<25 years) exhibit higher risk behaviors including smoking (AOR = 6.68, 95% CI:1.24–36.12, p = 0.03), reduced folic acid supplementation (AOR = 0.23, 95% CI:0.09–0.59, p = 0.002), and poorer health information access (AOR = 0.38, 95% CI:0.16–0.89, p = 0.03). ^7, 19^ PCC service utilization is further moderated by age, marital status, knowledge, and service accessibility. ^18^

Our analysis identified PCC knowledge (OR = 3.47, 95% CI:1.12-10.72; p = 0.03) and positive attitudes (OR = 2.96, 95% CI:1.96-4.46; p < 0.001) as the strongest behavioral predictors. These results align with Jordanian studies demonstrating significant knowledge-practice (p = 0.001) and attitude-practice (OR = 1.15, 95% CI:1.07-1.24) associations. ^29, 31^

Our study identified multiple significant factors influencing PCC practices: women aged >35 years showed 3.16-fold greater engagement (95% CI:1.82-5.49, p < 0.001), while higher education conferred 2.49-fold higher odds (95% CI:1.80-3.43, p < 0.001). Prior contraceptive use demonstrated a positive trend (OR = 3.43, 95% CI:0.88-13.38, p = 0.08), and notably, PCC counseling experience showed the strongest association with 9.41-fold improved practices (95% CI:4.96-17.84, p < 0.001). These findings collectively highlight key modifiable determinants for PCC improvement.

This study offers significant insights into the determinants of PCC knowledge, attitudes, and practices; however, several limitations must be acknowledged: the predominance of studies from Ethiopia may restrict generalizability to other contexts, and the cross-sectional design of the included studies precludes causal inferences. Moreover, variability in outcome measurements among research, especially with PCC behavior assessments, and possible reporting biases in primary studies may influence the reliability of aggregated findings. The omission of non-English publications and gray literature may also result in selection bias. These constraints underscore the necessity for further investigations employing longitudinal designs, uniform PCC measurements, and enhanced geographical diversity to fortify evidence-based recommendations.

## Conclusion

This study highlights significant gaps in PCC awareness and practices among reproductive-aged women, contributing to preventable maternal and infant health risks. Our findings demonstrate that educational attainment strongly predicts both PCC knowledge and positive attitudes, while improved knowledge directly enables better PCC practices. To address these gaps, we propose a three-pronged approach: (1) enhanced PCC service delivery through community-based education programs, (2) targeted initiatives to advance women’s educational opportunities, and (3) development of verified digital health resources and facility-based learning centers. These interventions should be healthcare professional-led to ensure medical accuracy and appropriate guidance in PCC counseling. The integration of these strategies can substantially strengthen PCC implementation and ultimately reduce preventable adverse pregnancy outcomes.

## Data Availability

No data are associated with this article. Supplementary materials are available in the supplementary data. Zenodo: [What Influences Women’s Knowledge, Attitudes, and Practices Toward Preconception Care?]. https://doi.org/10.5281/zenodo.15765388. ^32^ This project contains the following extended data:
• Supplementary eTable 1 Search Strategy 25622.docx
• Supplementary eTable 2 Critical Appraisal 25622.docx Data are available under the terms of the Creative Commons Attribution 4.0 International license (CC BY 4.0). Zenodo: PRISMA 2020 Checklist for ‘What Influences Women’s Knowledge, Attitudes, and Practices Toward Preconception Care? A Systematic Review and Meta-Analysis’. https://doi.org/10.5281/zenodo.15765388. ^32^ Data are available under the terms of the Creative Commons Attribution 4.0 International license (CC-BY 4.0). Software: Review Manager (RevMan version 5.3) is a free software tool for constructing systematic review and meta-analysis. To learn more, visit the link: https://www.cochrane.org/learn/courses-and-resources/software.
