# Supplementary material for: What Influences Women’s Knowledge, Attitudes, and Practices Toward Preconception Care? A Systematic Review and Meta-Analysis
Source: F1000Res. 2025 Sep 2;14:860. [Version 1] doi: 10.12688/f1000research.167200.1 (PMC12538205; doi:10.12688/f1000research.167200.1)
Supplement: Peer review history

# Reviewer report: Reviewer response for version 1

**DOI:** 10.5256/f1000research.184291.r420890

**Published:** 2025-11-22

**Authors:** Martha Irene Kartasurya

**Affiliations:** 1 Universitas Diponegoro, Semarang, Indonesia

This systematic review and meta analysis had been done rigorously on pre conception care. Not many studies had been done in this area. There were two databases used PubMed and Scopus for finding the articles. Thirteen cross sectional studies had been reviewed and before the review the critical appraisal had been done through JBI tool for cross sectional study. The procedures of PRISMA Guidelines had been followed. Meta analysis was done through RevMan3 and random effect models were used. The forest plots had been shown for KAP determinants of PCC. Many risk factors had been assessed. The results found that age, education levels, healthcare access and prior PCC engagement were important determinants of PCC. However, some limitation were acknowledged, including the heterogeneity of the studies, the variability of outcome measurements and the limitation of using cross sectional data. 

 Here are some suggestions to improve the manuscript.

 1. In the conclusion of the abstract it is written: Interventions targeting these determinants could improve preconception care uptake and maternal health outcomes. 

 Comment: determinants such as age can't be changed, therefore the sentence should be adjusted.

 2. In PubMed searching they were no "Risk factors" term used. 

 3. In Science direct searching the authors used: ((((women) AND (risk factors)) OR (knowledge)) OR (attitudes)) AND (practice)) AND (preconception care). For "practice" the Boolean search word should have been "OR", instead of  AND. 

 4. In the method section, after critical appraisal, was there any study excluded?

 5. In Table 1, in study by Demeke, et al, in findings column: "paritas" should be "parity". Similar typo was in study by Fikadu et al.

 6. In Figure 2, study by Demeke et al and Sori et al showed a very large Odd Ratio and Confidence interval. Is there any mistake in data input?

 7. The Titles of Figures 3 to 16 it is better to start with the determinants variable instead of the dependent variables. For example: Figure 3: Forest plot PCC knowledge and parity. It is better to changed into: Forest plots of parity on PCC knowledge.

 8. In the second sentence of discussion, it is stated that : "Our findings confirm that PCC is critical for reducing maternal and infant morbidity and mortality,20–23 requiring both multisectoral interventions24 and sustained government engagement.10"

 This systematic review did not confirm that PCC is critical for reducing maternal and infant morbidity and mortality and so on. Thus this sentence should be revised.

 9. There is no funnel plot presented to show the publication bias. Thus, it is better to mention in the method section, the reason why funnel plots are not presented.

Are the rationale for, and objectives of, the Systematic Review clearly stated?

Yes

Is the statistical analysis and its interpretation appropriate?

I cannot comment. A qualified statistician is required.

If this is a Living Systematic Review, is the ‘living’ method appropriate and is the search schedule clearly defined and justified? (‘Living Systematic Review’ or a variation of this term should be included in the title.)

Not applicable

Are sufficient details of the methods and analysis provided to allow replication by others?

Partly

Are the conclusions drawn adequately supported by the results presented in the review?

Yes

Reviewer Expertise:

maternal and child health

I confirm that I have read this submission and believe that I have an appropriate level of expertise to confirm that it is of an acceptable scientific standard.

## References

[1] : Model selection between the fixed-effects model and the random-effects model in meta-analysis. *Statistics and Its Interface* .2020;13(4) : 10.4310/SII.2020.v13.n4.a7 501-510 10.4310/SII.2020.v13.n4.a7

# Reviewer report: Reviewer response for version 1

**DOI:** 10.5256/f1000research.184291.r420891

**Published:** 2025-10-20

**Authors:** Irna Nursanti

**Affiliations:** 1 Universitas Muhammadiyah Jakarta, Jakarta Pusat, JAVA, Indonesia

This work provides a comprehensive and timely systematic review and meta-analysis that advances mother and child health. The authors synthesize 13 observational studies to identify and quantify the major determinants—such as education level, age, prior training, and counseling—that significantly affect women's preconception care knowledge, attitudes, and practices. The study follows PRISMA principles and uses a complete meta-analysis to find powerful, quantitative connections (e.g., a 28-fold increase in knowledge with greater education) that policymakers and healthcare practitioners may use. The clear, practical findings highlight PCC absorption mechanisms and provide a strong scientific framework for devising targeted interventions to overcome identified gaps, minimizing unnecessary maternal and newborn death.

Are the rationale for, and objectives of, the Systematic Review clearly stated?

Yes

Is the statistical analysis and its interpretation appropriate?

Yes

If this is a Living Systematic Review, is the ‘living’ method appropriate and is the search schedule clearly defined and justified? (‘Living Systematic Review’ or a variation of this term should be included in the title.)

No

Are sufficient details of the methods and analysis provided to allow replication by others?

Yes

Are the conclusions drawn adequately supported by the results presented in the review?

Yes

Reviewer Expertise:

Maternity Nursing

I confirm that I have read this submission and believe that I have an appropriate level of expertise to confirm that it is of an acceptable scientific standard.

# Response

**Published:** 2025-10-23

**Authors:** faizul hasan

**Affiliations:** faculty of nursing, Chulalongkorn University, Bangkok, Bangkok, Thailand

Thank you so much for this incredibly generous and thoughtful feedback on our work. We are truly delighted that you found our systematic review and meta-analysis to be comprehensive, timely, and a valuable contribution to mother and child health. Your recognition of the potential practical utility of our findings for policymakers and healthcare practitioners is especially rewarding and aligns perfectly with our hopes for this research.

# Reviewer report: Reviewer response for version 1

**DOI:** 10.5256/f1000research.184291.r420895

**Published:** 2025-10-20

**Authors:** Sri Sumarni

**Affiliations:** 1 Poltekkes Kemenkes Semarang, Semarang, Indonesia

This study is targeting determinants which might could improve PCC uptake and MHO. The result of this study involved 13 study. The critical risks lifestyle might be suggested for the next study, such as exercise, smoking, consuming herbal medicine, and any other. The method is sufficient to analyse data.  Prisma flow diagram is meaningful. **This study is worthy of indexing.**

Are the rationale for, and objectives of, the Systematic Review clearly stated?

Yes

Is the statistical analysis and its interpretation appropriate?

Yes

If this is a Living Systematic Review, is the ‘living’ method appropriate and is the search schedule clearly defined and justified? (‘Living Systematic Review’ or a variation of this term should be included in the title.)

Yes

Are sufficient details of the methods and analysis provided to allow replication by others?

Yes

Are the conclusions drawn adequately supported by the results presented in the review?

Yes

Reviewer Expertise:

Nursing, Midwifery, Woman and Teenage Reproductive Health

I confirm that I have read this submission and believe that I have an appropriate level of expertise to confirm that it is of an acceptable scientific standard.

# Response

**Published:** 2025-10-23

**Authors:** faizul hasan

**Affiliations:** faculty of nursing, Chulalongkorn University, Bangkok, Bangkok, Thailand

Thank you for your thoughtful review and positive feedback on our manuscript. We appreciate your acknowledgment of the methodology and PRISMA flow diagram. We also agree with your valuable suggestion to explore critical lifestyle risks like exercise, smoking, and herbal medicine in future studies to build upon our current findings.
